# Alteration of salivary microbiome in periodontitis with or without type-2 diabetes mellitus and metformin treatment

**DOI:** 10.1038/s41598-020-72035-1

**Published:** 2020-09-21

**Authors:** Xiaoyu Sun, Meihui Li, Li Xia, Zhaohui Fang, Shenjun Yu, Jike Gao, Qiang Feng, Pishan Yang

**Affiliations:** 1 Department of Periodontology, School and Hospital of Stomatology, Cheeloo College of Medicine, Shandong University & Shandong Key Laboratory of Oral Tissue Regeneration & Shandong Engineering Laboratory for Dental Materials and Oral Tissue Regeneration, Shandong University, Jinan, Shandong China; 2 Department of Human Microbiome, School and Hospital of Stomatology, Cheeloo College of Medicine, Shandong University & Shandong Key Laboratory of Oral Tissue Regeneration & Shandong Engineering Laboratory for Dental Materials and Oral Tissue Regeneration, Shandong University, Jinan, Shandong China; 3 Department of Periodontology, Stomatologic Hospital & College, Key Laboratory of Oral Diseases Research of Anhui Province, Anhui Medical University, Hefei, China; 4 Department of Endocrinology and Metabolism, The First Affiliated Hospital of Anhui Medical University, Hefei, China; 5 Department of Endocrine Disease, The First Affiliated Hospital of Anhui University of Chinese Medicine, Hefei, China; 6 Department of Periodontology, School of Stomatology, Shandong University, Jinan, Shandong China

**Keywords:** Applied microbiology, Clinical microbiology

## Abstract

We aimed to explore the effects of type-2 diabetes mellitus (T2DM) and hypoglycemic therapy on the salivary microbiome in periodontitis patients and identify the potential salivary micro-biomarker for the early warning of T2DM. Saliva samples were collected from healthy individuals (Health), periodontitis patients (P), T2DM patients, periodontitis patients with T2DM (DAP), and DAP patients treated with Metformin (Met). Samples were determined by16S rRNA gene sequencing. 29 phyla, 322 genera, and 333 species of salivary microbiome were annotated. Compared to the Health group, the P and DAP group showed a significantly higher diversity of saliva microbiota, while the T2DM and Met group had no significant difference in microbial abundance but showed a trend of increasing diversity. Other than well-known periodontitis-inducing pathogens, the proportion of *Prevotella copri*, *Alloprevotella rava*, and *Ralstonia pickettii*, etc. were also significantly increased in periodontitis patients with or without T2DM. After effective glycemic control, the abundance of *Prevotella copri*, *Alloprevotella rava*, *Ralstonia pickettii*, etc. decreased in periodontitis patients with companion T2DM. The accuracies of the classification models in differentiating Health-vs.-P, DAP-vs.-P, and T2DM-vs.-P were 100%, 96.3%, and 98.1%, respectively. Hypoglycemic therapy could reconstruct the saliva microbiota and hence improve the localized conditions of diabetes patients with periodontitis.

## Introduction

Periodontitis is one of the most frequent bacteria-induced inflammatory diseases in the oral cavity. The latest survey by the Chinese Stomatological Association reported the rate of healthy periodontal tissue to be only 5% in the 55–64 year old group^1^. Periodontitis is the major cause of tooth loss in adults, and it seriously affects mastication and facial aesthetics. In addition, periodontitis also associates with a group of systemic diseases, including cardio-/cerebro-vascular diseases, tumors, type 2 diabetes mellitus (T2DM), pregnancy complications, and rheumatoid arthritis^2–4^. Studies have reported the bilateral relationship between T2DM and periodontitis, as individuals with diabetes are more susceptible to periodontitis than those with normal blood glucose levels due to the secondary changes in the oral environment, and the obliteration of tissues and supporting bones, as a consequence of T2DM, may cause rigorous or progressive periodontitis^5–9^. Periodontal inflammation can affect the level of glycosylated hemoglobin (HbA1c) and subsequently result in pre-diabetes^10^. Teeuw et al. revealed that HbA1c levels of T2DM patients decreased by 0.4% after periodontal therapy, which may have been due to the decrease in the secretion of local inflammatory factors, such as IL-6, CRP, MMP, and TNF-α, into the systemic circulation^11^. Active periodontal therapy can control inflammation of periodontal tissue, which might contribute to glycemic control^12^.

The oral microbiome is known to vary in response to oral and systemic diseases^13^. Studies have reported that several oral microbes are closely linked to T2DM. For example, the presence of *Porphyromonas gingivalis* with type II fimbriae in the periodontal pockets affected the glycemic index of individuals with T2DM, and poor glycemic control associates with increased proportions of red-complex microbes (*Porphyromonas gingivalis*, *Tannerella forsythia*, and *Treponema denticola*) in subgingival plaque samples^14–16^. Anbalagan R, et al. found that the relative proportions of *Streptococcus*, *Veillonella*, *Neisseria*, *Rothia*, *Actinomycetes*, *Fusobacterium*, and *Pigmentiphaga* in the T2DM group were similar to those of the healthy group^17^. Long et al. also analyzed the oral microbiome of T2DM patients and discovered that the relative abundance of *Actinobacteria*, which associates with a lower risk of developing T2DM, decreased^18^. Meanwhile, the relative proportions of *Treponema denticola*, *Prevotella nigrescens*, *Streptococcus sanguis*, *Streptococcus oralis*, and *Streptococcus intermadius* increased in supragingival plaque samples of T2DM patients. A significant difference in the number of *Streptococcus mutans* and *lactobacillus* was also observed between the saliva of T2DM and non-T2DM individuals^19^. These findings suggest that T2DM patients possess a unique oral microbial composition, suggesting that the oral microbiome contains information on potential biomarkers for predicting the risk of developing T2DM and for establishing early diagnostic models.

The salivary microbiome, which consists of microbes from the entire oral cavity, is easy to access and can be a potential source for predicting oral health^20^. Moreover, the salivary microbiome is very stable and not easily influenced by environmental factors^21^. In this study, we characterized the salivary microbial composition of patients with periodontitis and T2DM. We also evaluated the effect of hypoglycemic therapy and identified a group of highly effective biomarkers, which lays the foundation for further understanding the influence of periodontitis and diabetes on the salivary flora and the rapid identification of salivary markers for oral health.

## Results

### Clinical characteristics among different groups

The demographic data of the study population are listed in Table 1. Patients with periodontitis, including those in P, DAP, and Met groups, had significantly higher probing depth (PD), clinical attachment loss (CAL), bleeding index (BI), gingival index (GI), and plaque index (PLI) values than patients in T2DM and Health groups (*P* < 0.001). T2DM and DAP groups showed significantly higher BMI, HbA1c, and fasting plasma glucose levels than Health and P groups (*P* < 0.001). HbA1c and fasting plasma glucose levels in the Met group significantly decreased compared to T2DM and DAP groups (*P* < 0.001).

**Table 1:** Demographic data of the study population.

Parameter	DAP (n = 46)	Met (n = 20)	P (n = 31)	T2DM (n = 9)	Health (n = 27)
**Gender, n (%)**					
Male	17 (37.0)	12 (60.0)	10 (32.3)	3 (33.3)	7 (25.9)
Female	29 (63.0)	8 (40.0)	21 (67.7)	6 (66.7)	20 (74.1)
Age, years	60.98 ± 9.12**^ΔΔ^	59.25 ± 8.61**^ΔΔ^	44.55 ± 14.04**	44.67 ± 10.75	28.78 ± 7.51
**DM-related parameters**					
BMI (kg/m^2^)	25.21 ± 3.75**^ΔΔ^	25.86 ± 3.19**^ΔΔ^	23.16 ± 3.25*	27.59 ± 1.80	21.13 ± 2.43
HbA1C (%)	9.24 ± 1.37**^ΔΔ⋄⋄^	5.33 ± 0.53 **^ΔΔ^	4.86 ± 0.62	8.43 ± 1.41	5.06 ± 0.52
GLU (mmol/L)	9.79 ± 2.05**^ΔΔ⋄⋄^	6.18 ± 0.38**^ΔΔ^	5.77 ± 0.77	11.66 ± 1.87	5.18 ± 0.36
**Perio-related parameters**					
PD (mm)	6.46 ± 0.96**	6.00 ± 0.65**	6.42 ± 0.89**	2.11 ± 0.78	2.11 ± 0.42
BI	3.2 ± 0.58**^⋄⋄^	2.55 ± 0.51**^ΔΔ^	3.16 ± 0.58**	1.11 ± 0.33	0.52 ± 0.51
GI	1.87 ± 0.62**^ΔΔ^	2.00 ± 0.56**^Δ^	2.42 ± 0.50**	1.00 ± 0.50	0.44 ± 0.51
PLI	2.24 ± 0.60**	2.45 ± 0.51**	2.45 ± 0.57**	1.11 ± 0.33	1.04 ± 0.34
CAL (mm)	4.41 ± 1.11**	4.10 ± 0.72**^Δ^	4.65 ± 0.91**	0	0

Parameters including age, BMI, HbA1c, GLU, PD, CAL, BI, GI, and PLI are presented as mean ± standard deviation. Compared to the Health group, * represents *P* < 0.05, ** represents *P* < 0.01; compared to the P group, Δ represents *P* < 0.05, ΔΔ represents *P* < 0.01; compared to the Met group, ⋄ represents *P* < 0.05, ⋄⋄ represents *P* < 0.01. To be specific, the *P*-values for BMI were 0.009 for DAP versus P, 0.005 for Met versus P, and 0.02 for P versus Health; the *P*-value for GI was 0.010 for Met versus P; the *P*-value for CAL was 0.030 for Met versus P. All other unspecified *P*-values were less than 0.001.

We performed independent t-tests to compare the difference in clinical factors between groups (Table 1), except for the T2DM group which had a small sample size. For CAL, a comparison was performed among P, Met, and DAP groups. Compared to the P group, the Met group had significantly lower BI levels (*P* < 0.001), whereas the DAP group did not. HbA1C and GLU levels were significantly higher in the DAP group compared to the Met and Health groups (*P* < 0.001), whereas the Met group showed similar HbA1C levels to the Health group. After comparison of the DAP group to the P group, the P group showed significantly lower BMI, HbA1C, GLU, and GI levels (*P* < 0.001).

### Characteristics of microbial communities in different groups

**Figure 1:**
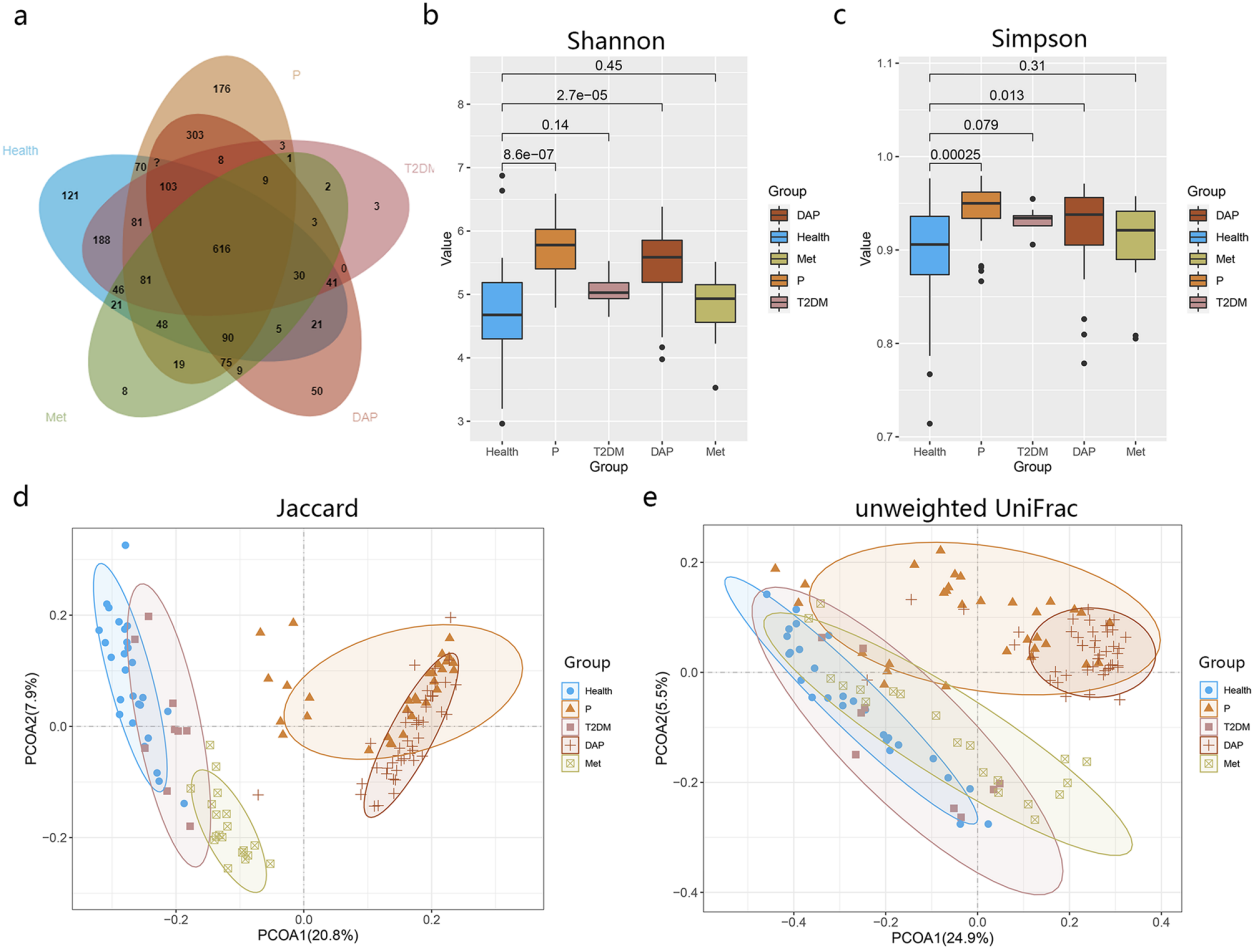
Phylogenic diversity of oral microbiota in different groups. (**a**) Venn diagram showing the number of shared OTUs between groups. Box plot showing the alpha-diversity of each group according to the (**b**) Shannon index and (**c**) Simpson index. The vertical lines indicate the range; the middle thick lines represent the median value; the boxes represent the upper and lower quartile values. The Wilcoxon rank-sum test was used to compare the Shannon index of the disease groups with the Health group. The beta-diversity of each group as shown by (**d**) Jaccard and (**e**) unweighted UniFrac in PCoA.

As described in the Materials and Methods, more than 9.08 Gb high-quality reads were obtained from 133 saliva samples. After filtering with a maxEE value of 1.5 for quality control, 8,345,256 clean reads were obtained, with each covering 58,147 reads on average. After clustering all of the qualified sequences at 97% similarity, we generated 2,331 operational taxonomic units (OTUs), of which 616 OTUs were shared by the healthy and disease groups (Fig. 1a). All OTUs were annotated into a total of 29 phyla, 60 classes, 97 orders, 175 families, 322 genera, and 333 species.

Next, we compared the microbial communities of different groups. At the phylum level, the predominant phyla of each group were *Firmicutes*, *Proteobacteria*, *Bacteroidetes*, *Fusobacteria*, and *Actinobacteria*, etc. The proportion of *Proteobacteria* was higher in the Health group compared to the disease groups, whereas that of *Bacteroidetes* was higher in the disease groups (highest in the P group) than in the healthy group. At the genus level, the predominant genera of each group included *Streptococcus*, *Prevotella*, *Neisseria*, *Fusobacterium*, *Rothia*, *Porphyromonas*, and *Treponema*, etc. The proportion of *Prevotella* was the lowest, whereas that of *Streptococcus* was the highest in the Health group. Opposite findings were observed for the disease groups. The proportions of *Porphyromonas* and *Treponema* were higher in both P and DAP groups and lower in Health and T2DM groups. At the species level, *Streptococcus dentisani*, *Haemophilus parainfluenzae*, *Prevotella copri*, *Porphyromonas endodontalis*, *Treponema medium*, and *Porphyromonas gingivalis*, etc. were the most abundant microbes. The proportion of *Streptococcus dentisani* was highest in the Health and Met groups, whereas it was lowest in the P group. These indicated that glycemic control induced the proportion of healthy condition-associated bacteria. The proportion of *Prevotella copri* was relatively higher in P and DAP groups and extremely low in the other groups, indicating that it closely associated with periodontitis-related conditions. Detailed results at each level were shown in Supplementary Fig. S1a–d online.

Alpha diversity, as reflected by the Shannon and Simpson indices, showed that the microbial diversity in P and DAP groups was significantly increased compared to the healthy group, and there was no significant difference between T2DM and Met (Fig. 1b,c), indicating that the relative abundance of the oral microbiomes changed in response to the periodontal status and blood sugar status.

Beta-diversity was significantly different among the groups. In PCoA, the Jaccard distance showed a remarkable shift in the microbial distribution among the five groups (Fig. 1d). The PCoA results of the unweighted UniFrac distance and the Jaccard distance also showed a similar pattern (Fig. 1e), but the healthy and disease groups displayed a better classification efficiency for the Jaccard distance. In PCoA, a significant difference was identified between the population with periodontitis and abnormal blood glucose status (i.e., P, DAP, and T2DM groups) and the population with well-controlled blood glucose status (i.e., Health and Met groups).

Thereafter, we analyzed the common “core microbiomes” among the five groups. At the phylum level, seven phyla of common “core microbiomes” were found, including *Spirochaetes*, *Firmicutes*, and *Proteobacteria*, etc. At the genus level, thirty-seven common “core microbiomes” were found, including *Streptococcus*, *Prevotella*, *Porphyromonas*, and *Treponema*, etc. Of the shared “core microbiomes”, the contents of T2DM and Met groups were consistent with those of the Health group, whereas the contents of DAP and P groups were generally higher than those of the Health group. At the species level, forty-eight types of common “core microbiomes” were found, including *Streptococcus dentisani*, *Porphyromonas endodontalis*, *Porphyromonas gingivalis*, *Treponema medium*, and *Prevotella intermedia*. The distribution pattern at the species level was similar to that at the genus level (Supplementary Fig. S2a–c online).

### Microbial variations associated with periodontitis and T2DM

To investigate the impact of periodontitis on the salivary microbiome, we examined changes in the salivary microbiome between disease and Health groups, between P/T2DM groups and the DAP group, and changes in the saliva microbiota after glycemic control with metformin.

**Figure 2:**
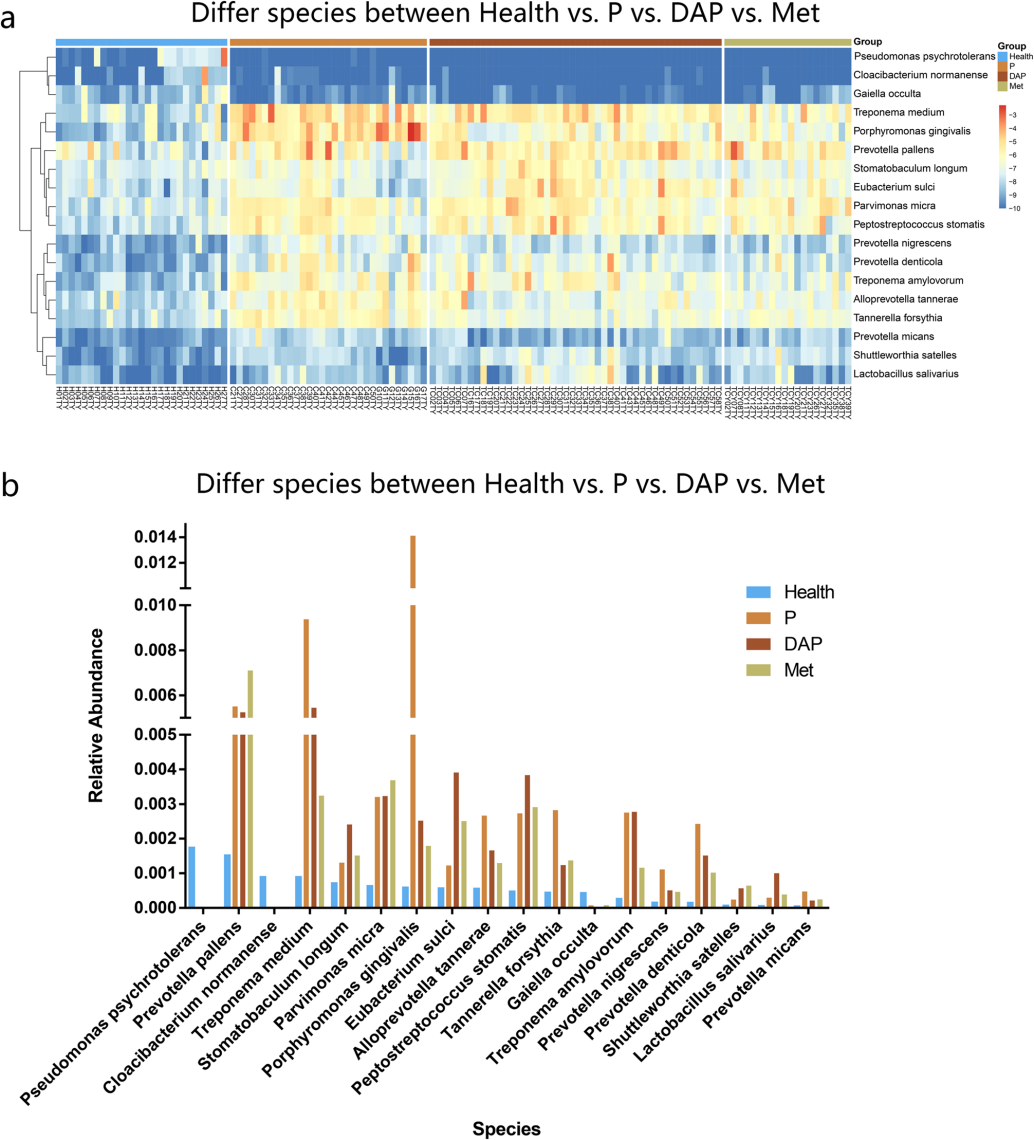
Relative abundance of microbial communities in different groups. (**a**) Heatmap demonstrating the relative abundance of microbial communities in P, DAP, Met, and Health groups. (**b**) Bar chart comparing the relative abundance of microbial communities in P, DAP, Met, and Health groups.

Firstly, we compared each disease group respectively with the Health group. Due to the small sample size and high similarity with the Health group in terms of alpha-diversity, the T2DM group was not used in the subsequent comparison. Except for the T2DM group, bacteria with a difference in the relative abundance between each disease group and the Health group were listed in Fig. 2a. The proportions of *Porphyromonas gingivalis*, *Treponema medium*, *Tannerella forsythia*, *Parvimonas micra*, *Prevotella pallens*, and *Peptostreptococcus stomatis* were generally higher in disease groups compared to the Health group. Meanwhile, *Pseudomonas psychrotolerans*, *Cloacibaterium normanense*, and *Gaiella occulta* were enriched in the Health group.

Next, we analyzed all the disease groups with microbiota having a more than three times difference in the relative abundance compared to the Health group (Fig. 2b). Compared to the disease groups, the Health group displayed a relatively higher proportion of *Pseudomonas psychrotolerans* and *Cloacibacterium normanense*, whereas the disease groups had relatively higher proportions of *Prevotella pallens*, *Treponema medium*, *Parvimonas micra*, *Porphyromonas gingivalis*, *Prevotella nigrescens*, and *Tannerella forsythia*, etc.

**Figure 3:**
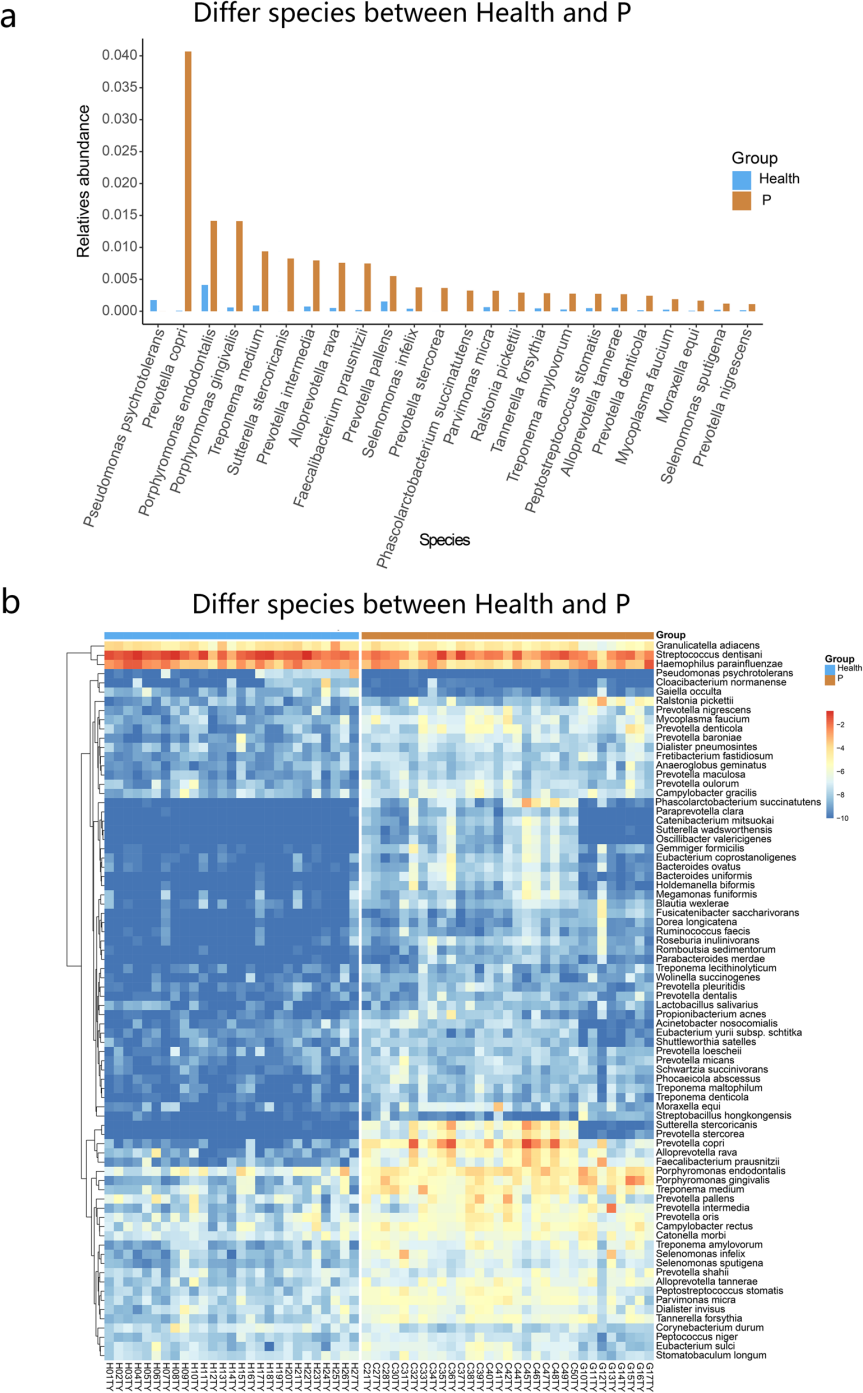
Comparison of the relative abundance between P and Health groups. (**a**) Bar chart showing the relative abundance of salivary microbes with a difference greater than 10^–4^ between P and Health groups. (**b**) Heatmap showing the difference in relative abundance of salivary microbes between P and Health groups.

To investigate the impact of periodontitis on the salivary microbiome, we further compared the difference in microbiota between Health and P groups at phylum, genus, species, and OTU levels. At the species level, the P group showed a significant difference in the salivary microbial composition compared to the Health group. The proportions of *Prevotella copri*, *Porphyromonas endodontalis*, *Porphyromonas gingivalis*, *Treponema medium*, *Tannerella forsythia*, *Prevotella nigrescens*, and *Prevotella intermedia*, etc. were higher in the P group, whereas the proportion of *Pseudomonas psychrotolerans* was higher in the Health group (Fig. 3). Among them, *Porphyromonas gingivalis* and *Tannerella forsythia* are well-known members of the red-complex bacterial family, whereas *Prevotella nigrescens* and *Prevotella intermedia* are well-known members of the orange-complex bacterial family. These bacteria closely associate with inflammation of periodontal tissue. Furthermore, we found significant differences in abundance of *Prevotella copri*, *Porphyromonas endodontalis*, *Treponema medium*, *Alloprevotella rava*, *Sutterella intermedia*, *Treponema amylovorum*, and *Faecalibacterium prausnitzii*, etc. between the two groups.

**Figure 4:**
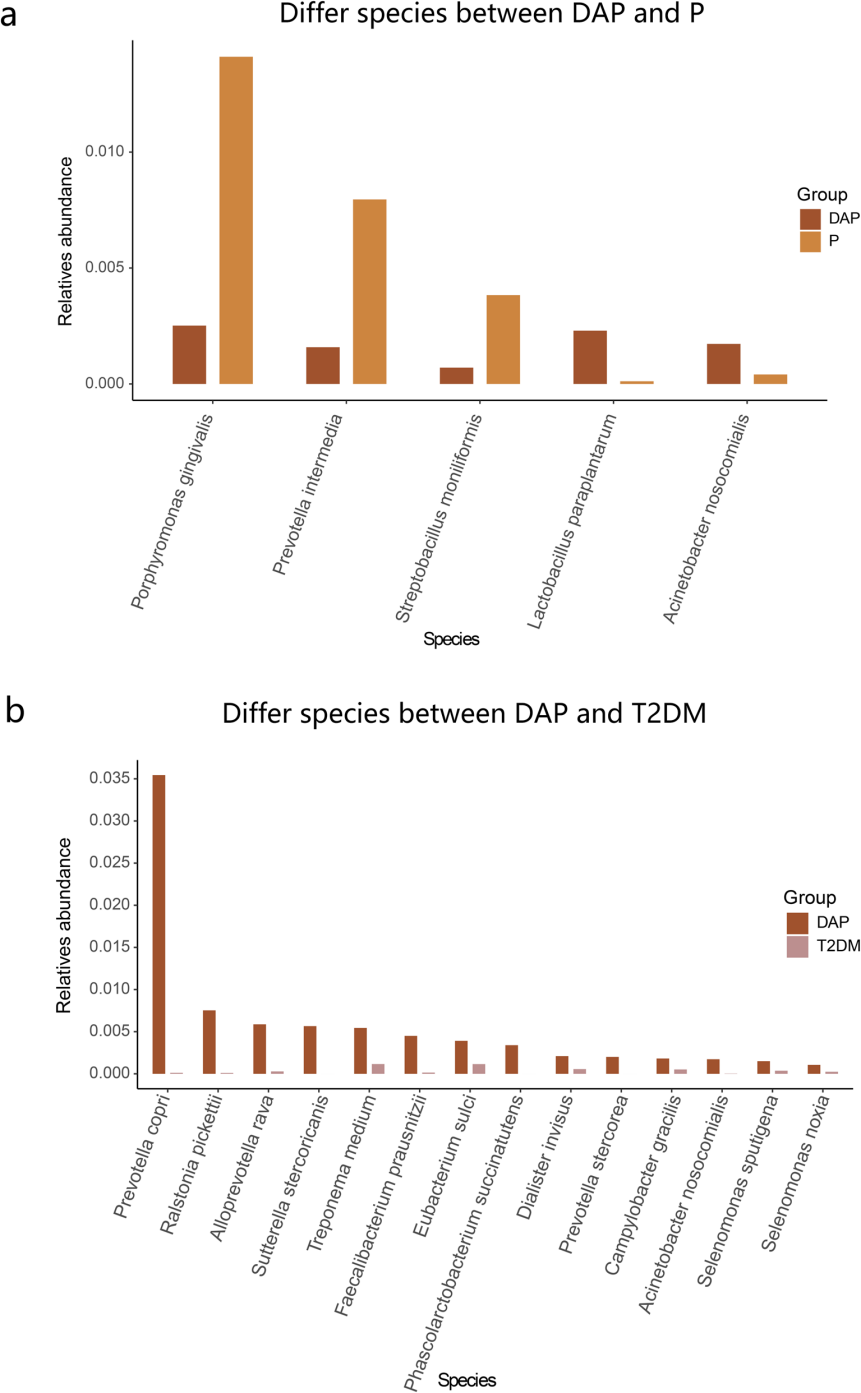
Comparison of the relative abundance among DAP, P, and T2DM groups. Bar chart showing the relative abundance of salivary microbes between (**a**) DAP and P groups, and (**b**) T2DM and DAP groups.

To further investigate the effect of the interaction between T2DM and periodontitis on changes in the salivary microbiome, we compared the difference in relative abundance among the DAP, P, and T2DM groups. After comparison of the DAP group to the P group (Fig. 4a), the proportions of *Lactobacillus paraplantarum* and *Acinetobacter nosocomialis* were relatively higher in the DAP group, whereas the proportions of *Porphyromonas gingivalis*, *Prevotella intermedia*, and *Streptobacillus moniliformis* were relatively higher in the P group. These findings implied that *Lactobacillus paraplantarum* and *Acinetobacter nosocomialis* were associated with the synergistic pathogenic effect of microbiota under high glucose conditions. Furthermore, the DAP group had relatively higher proportions of *Prevotella copri*, *Ralstonia pickettii*, *Alloprevotella rava*, *Treponema medium*, *Faecalibacterium prausnitzii*, *Eubacterium sulci*, and *Acinetobacter nosocomialis*, etc. compared to the T2DM group (Fig. 4b).

**Figure 5:**
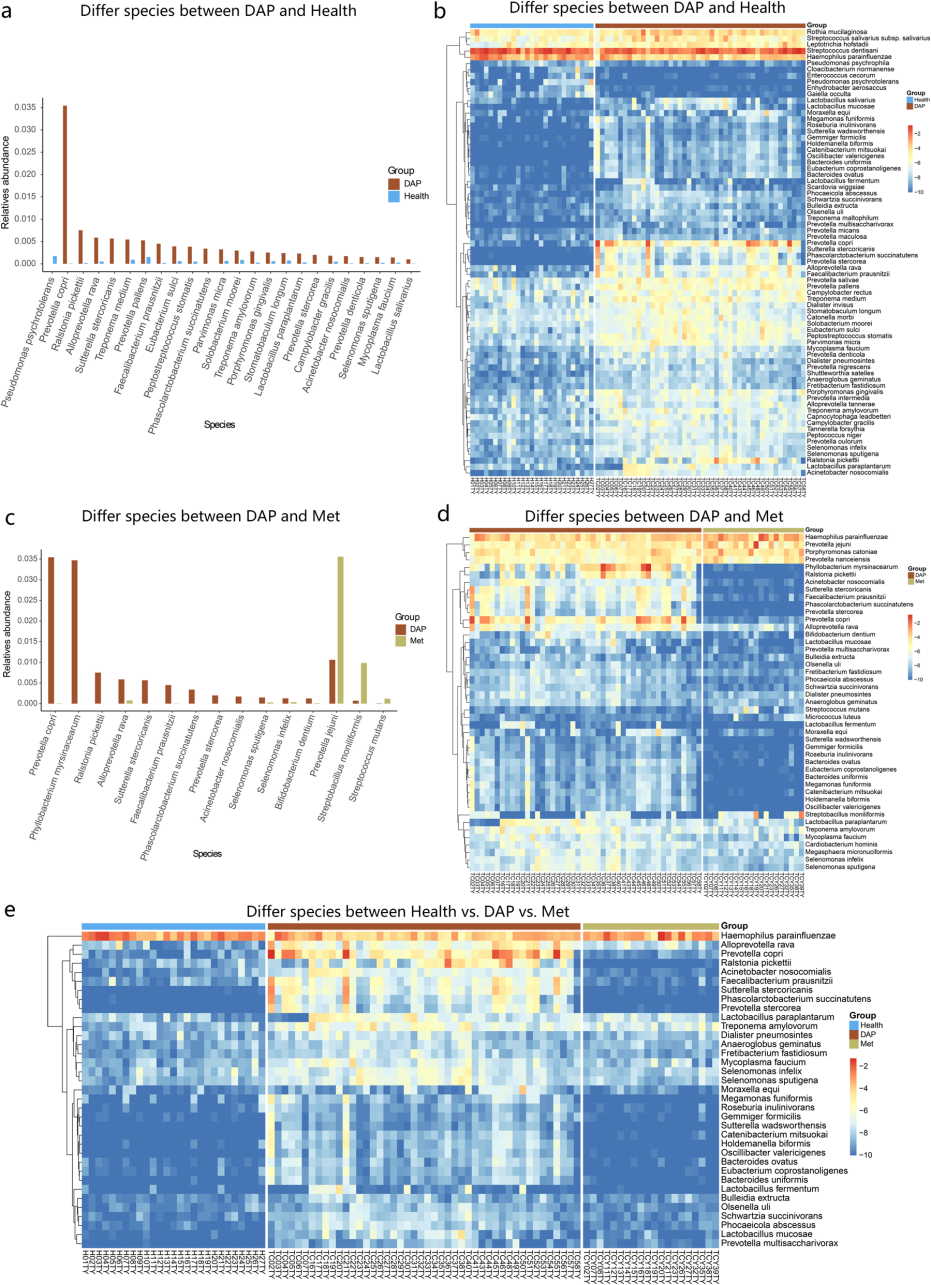
Comparison of the relative abundance between groups. Bar chart showing the relative abundance of salivary microbes in (**a**) DAP and Health groups, and (**c**) DAP and Met groups. Heatmap showing the difference in relative abundance of salivary microbes between (**b**) DAP and Health groups, and (**d**) DAP and Met groups. (**e**) Heatmap showing the different bacteria in Health vs. DAP groups and DAP vs. Met groups.

Lastly, to examine the effect of glycemic control on the salivary microbiome of DAP patients, we compared the relative abundance of salivary microbes among DAP, Met, and Health groups. For the DAP group (patients without active glycemic control), an increase in the relative proportions of *Prevotella copri*, *Rlastonia pickettii*, *Alloprevotella rava*, *Treponema medium*, *Prevotella pallens*, *Peptostreptococcus stomatis*, *Parvimonas micra*, *Solobacterium moorei*, *Porphyromonas gingivalis*, *Lactobacillus paraplantarum*, and *Lactobacilus salivarius* was observed (Fig. 5a,b). After comparison of the DAP group to the Met group (Fig. 5c,d), the proportions of *Prevotella copri*, *Alloprevotella rava*, *Phyllobacterium myrsinacearum*, and *Ralstonia pickettii* were significantly higher in the DAP group, whereas the proportions of *Streptobacillus moniliformis*, *Streptococcus mutans*, and *Prevotella jejuni* were significantly higher in the Met group (patients were under glycemic control). The differences in the relative proportions of bacteria of “Health vs. DAP groups” and “DAP vs. Met groups” were plotted in a heatmap (Fig. 5e). As shown in the figure, the distribution patterns of Met group and Health groups were similar. The proportions of *Prevotella copri*, *Rlastonia pickettii*, *Alloprevotella rava*, and *Sutterella Stercoricanis* , etc. were significantly higher in the DAP group compared to Met and Health groups (Supplementary Fig. S3a online), suggesting that after effective glycemic control, the salivary microbial composition of periodontitis patients with companion T2DM resembled that of healthy individuals. After comparison to the Health group, the Met group also showed significant differences in the relative proportions of *Prevotella jejuni*, *Prevotella salivae*, *Prevotella pallens*, *Parvimonas micra*, *Prevotella denticola*, and *Treponema medium*, as well as *Peptostreptococcus stomatis*, *Treponema amylovorum*,and *Solobacterium moorei* (Supplementary Fig. S3b, c online). Supplementary Fig. S3d online lists the differential bacteria of “Health vs. DAP groups” and “Health vs. Met groups” by pair-wise comparison of the three groups. The results revealed that the relative abundance of *Porphyromonas gingivalis*, *Treponema medium*, *Eubaterium sulci*, *Lactobacillus paraplantarum*, and *Lactobacillus salivarius*, etc. decreased after effective glycemic control.

### Relationship between microbial communities and clinical parameters

**Figure 6:**
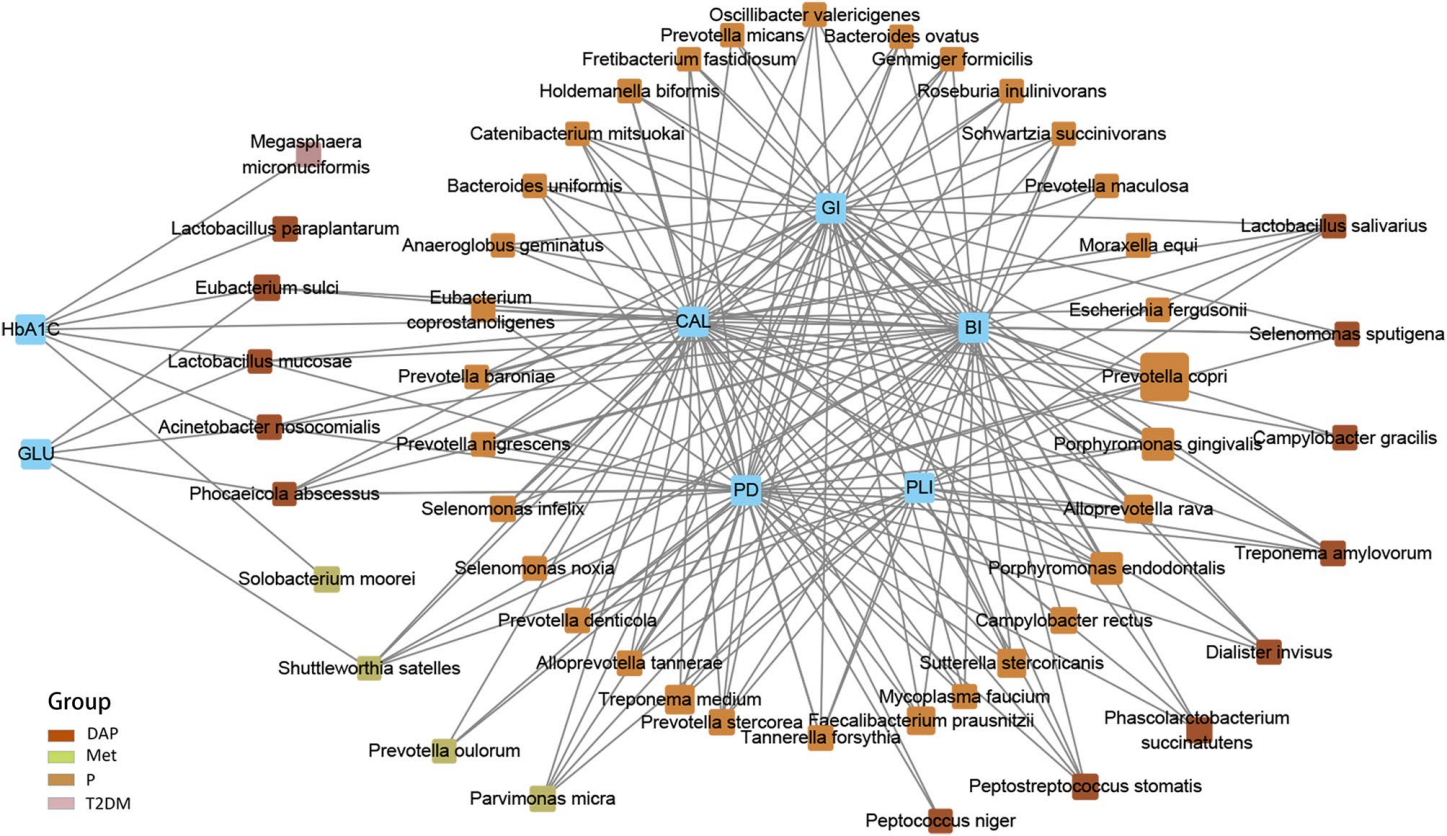
Association between microbial communities and clinical parameters. Graphical illustration of the correlation between microbial communities and clinical parameters. Microbiota were divided into corresponding groups according to the highest relative abundance, as indicated by differences in box color in the graph. The size of the colored box represents the relative abundance of that microbe in that group. A line indicates the two factors were positively associated (*P* < 0.05 in independent t-test).

In order to clarify the relationship between the changes in the salivary microbiome and periodontitis/T2DM, we analyzed the correlations between clinical parameters with different microbes. The results showed that a group of clinical parameters were positively associated with a group of microbes with a correlation value of ≥ 0.4 (Fig. 6). Periodontitis-related parameters (i.e., PD, CAL, BI, GI, and PLI) were positively correlated with several well-known periodontitis-inducing microbiota, such as *Porphyromonas gingivalis* and *Tannerella forsythia*, as well as *Prevotella copri*, *Alloprevotella rava*, *Prevotella denticola*, *Catenibacterium mitsuokai*, *Prevotella stercorea*, and *Treponema medium*, etc. *Prevotella copri* showed the highest relative abundance in the P group and closely associated with periodontitis. Furthermore, DM-related parameters were positively correlated with *Megaspaera micronuciformis*, *Eubacterium sulci*, *Lactobacilus mucosae*, *Lactobacilus paraplantarum*, *Phocaecola abscessus*, *Acinetobacter nosocomialis*, *Solobaterium moorei*, and*Shuttleworthia satelles*. Among them, only *Megasphaera micronuciformis*, *Lactobacilus paraplantarum*, and *Solobacterium moorei* were correlated with HbA1c. The proportions of *Solobaterium moorei* and *Shuttleworthia satelles*, which were relatively higher in the Met group, correlated with GLU and HbA1c. These findings revealed that the oral environment was the major factor affecting the oral microbial communities in the studied disease groups. The salivary microbiomes were positively associated with each other (Supplementary Fig. S4a online). The results with correlation values of ≥ 0.5 and ≥ 0.6 were are shown in Supplementary Fig. S4b and c online, respectively.

### Predicting diseases with different microbes

**Figure 7:**
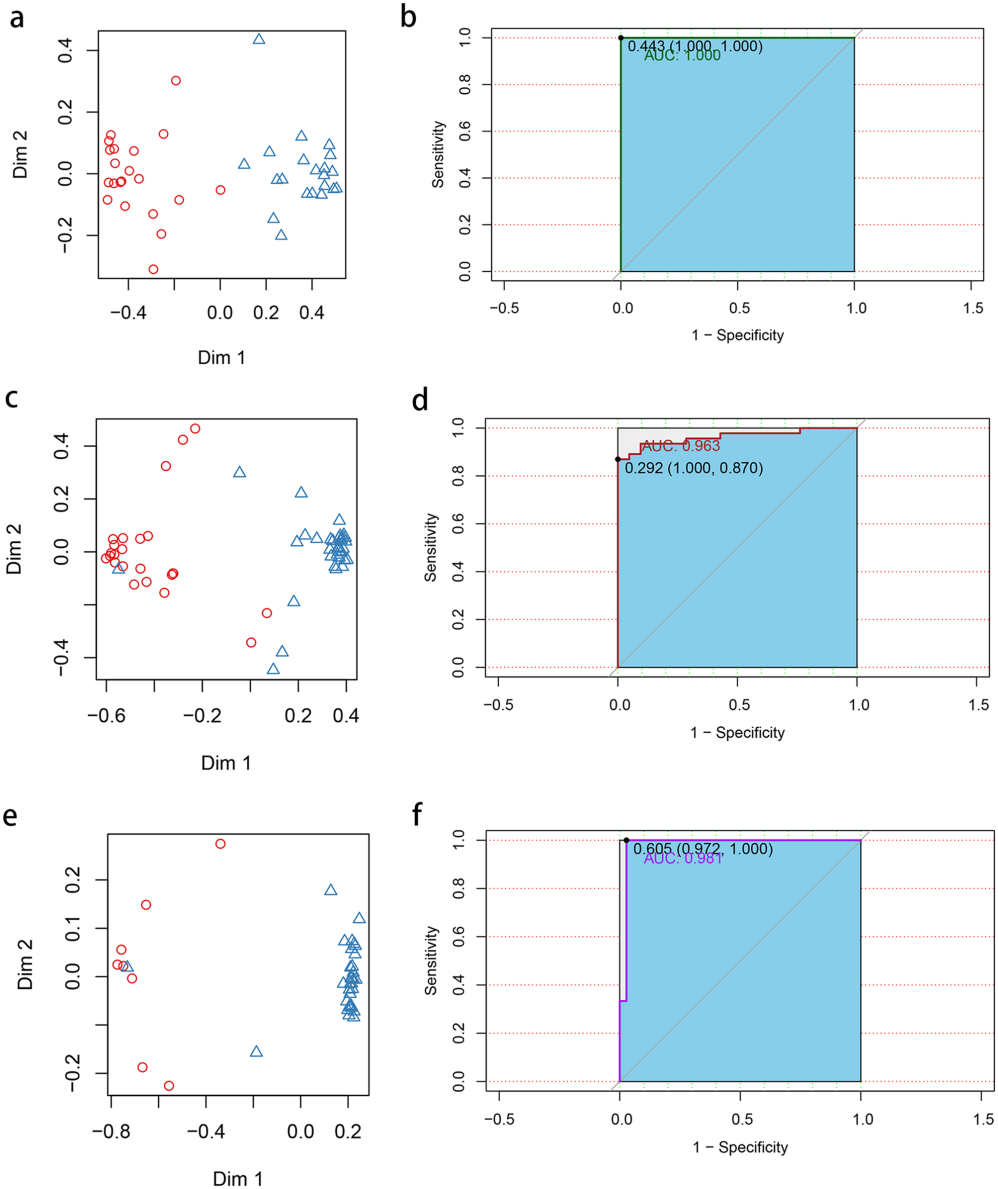
Prediction performance of the random forest models. (**a**) Two-dimensional dot plot showing the distribution of the P group vs. the Health group. (**b**) ROC curve of the selected core microbiota in differentiating between the P group and the Health group. (**c**) Two-dimensional dot plot showing the distribution of the P group vs. the DAP group. (**d**) ROC curve of the selected core microbiota in differentiating between the P group and the DAP group. (**e**) Two-dimensional dot plot showing the distribution of the T2DM group vs. the DAP group. (**f**) ROC curve of the selected core microbiota in differentiating between the T2DM group and the DAP group.

To examine the prediction efficiency, different microbes were selected as salivary micro-biomarkers by random forest models. To differentiate the Health group from the P group, the top 15 most important different microbes, including *Faecalibacterium prausnitzii*, *Prevotella copri*, *Porphyromonas gingivalis*, and *Phocaeicola abscessus*, etc. were used to train the model (Supplementary Fig. S4a online). The optimal cutoff threshold was 0.443, and the classification accuracy was 100% (AUC = 1.000; Fig. 7a,b). To differentiate the P group from the DAP group, *Lactobacillus paraplantarum*, *Granulicatella elegans*, *Porphyromonas gingivalis*, *Rothia mucilaginosa*, and *Prevotella salivae* , etc. were used to train the model (Supplementary Fig. S4b online). The optimal cutoff threshold was 0.292, and the classification accuracy was 96.3% (AUC = 0.963; Fig. 7c,d). To differentiate the T2DM group from the DAP group, *Sutterella stercoricanis*, *Alloprevotella rava*, *Prevotellacopri*, *Treponema medium*, *Faecalibacterium prausnitzii*, *Eubacterium sulci*, and *Acinetobacter nosocomialis*, etc. were used to train the model (Supplementary Fig. S4c online). The optimal cutoff threshold was 0.605, and the classification accuracy was 98.1% (AUC = 0.981; Fig. 7e,f).

Due to the small sample size of the T2DM group, we found a recent paper that consists of patients with highly similar inclusion criteria with our T2DM and DAP group and has its sequencing data of saliva samples available to serve as a validation set to validate the reliability of the group data^22^. High quality reads with a sequencing depth of 30,000 was screened and a final validation set consisting of 16 T2DM patients and 15 DAP patients was obtained. 18 of the 38 differential bacteria including *Alloprevotella rava* and *Porphyromonas endodontalis* were also present in the validation set. With the above 18 microbiota, the accuracy of the classification model was 82.2% in the validation set (AUC = 0.822; Supplementary Fig. S6).

## Discussion

The recent advances in the pathogenesis of periodontal diseases have led to more and more evidence supporting that periodontal inflammation is caused by multi-bacterial infection and the imbalance of periodontal environment associated with the abnormal bacterial population in the periodontal pocket. Moreover, studies discovered that DM is closely associated with periodontitis. The hyperglycemic condition can lead to impairment of the host’s immune functions, inhibit the bone formation, and enhance the release of cytokines such as interleukin-1β and -6^23,24^. Despite the progressive development of high-throughput sequencing techniques, there are still a limited number of studies investigating the oral microbial composition of periodontitis patients with companion T2DM. Saliva samples are complicated, comprising urea, ammonia, uric acid, glucose, cholesterol, fatty acid, as well as over 700 species of microorganisms that are related to oral and systemic diseases^25^. Salivary tests can be readily carried out with low processing costs and fast turnaround time^26^. In the present study, we demonstrated that T2DM and periodontitis patients possess unique saliva microbial communities. Moreover, we found that blood sugar status has a remarkable effect on the saliva microbial composition in patients with periodontitis. The abnormal blood sugar status may induce an increase in both the microbiota diversity and relative abundance of the disease-related microbiota. Interestingly, the combined effect of T2DM and periodontitis on the change of saliva microbial composition was significantly greater than that of T2DM alone, suggesting that periodontitis-related parameters are the main factors influencing the saliva microbial composition. Classification models established with the selected core microbiota had superior efficiencies in differentiating different diseases.

Metformin as a first-line treatment option for T2DM shows its advantages in terms of safety, effectiveness, low cost, and lowering the chance of cardiovascular adverse effects. It has been widely applied in the clinical treatment of T2DM for over 60 years^27^. A recent study suggested that metformin could improve the gut microbiome of T2DM patients and enhance one’s hypoglycemic effect^28^. All the disease groups displayed higher microbiota diversity (not conclusive for the T2DM group) and different patterns of microbial communities compared to the Health group, except for the Met group. Several previous reports also disclosed consistent results^17,22,29^. Generally speaking, the diversity and the relative abundance of oral bacteria increase in periodontitis patients with or without type-2 diabetes mellitus. The Met group resembled the Health group in terms of microbiota diversity and abundance. These suggested that the glycemic control effect of the metformin treatment in DAP patients may influence the oral microbial composition, and lead to a decrease in the relative proportion of disease-related microbiota. Also, this shed light on the claim that systemic changes may play a role in affecting the conditions of the localized microenvironment.

Compared to the Health group, the P group showed a more complex microbial diversity. Well-proven periodontitis-causing microbiota including *Porphyromonas gingivalis*, *Prevotella intermedia*, *Prevotella nigrescens* and *Tannerella forsythia* were identified as predominant species in the P group, in which *Porphyromonas gingivalis* and *Tannerella forsythia* are red-complex bacteria, while *Prevotellaintermedia* and *Prevotella nigrescens* are orange-complex bacteria. The two types of bacteria showed a significant synergistic effect. We also discovered from our results that *Prevotella copri* was associated with periodontitis-related conditions. When combining the P group and DAP group, *Lactobacillus paraplantarum* and *Acinetobacter nosocomialis* showed significantly higher relative abundance, indicating that these bacterial species were associated with blood sugar status.

Li et al. also reported that the oral bacterial compositions were similar in T2DM patients and healthy individuals, except that the relative abundance of *Bulleidia*, *Ruminoco- ccaceae*, and *Helicobacter pylori* were higher in the T2DM patients^30^. In agreement with the above studies, our results indicated that the T2DM group showed no statistically significant difference compared to the Health group, but a trend of relatively higher microbial diversity was observed. This could be due to the small sample size of the T2DM group (n = 9), which leads to bias. Also, this may be explained by the weak association of T2DM-related clinical parameters with the saliva microbial composition. The effect of T2DM on saliva microbial composition may only be significant when co-existing with periodontitis, although a further study with more T2DM patients is required to validate this claim.

It should be noted that different populations with different diet and living habits could possess a different saliva microbial composition. Takeshita et al. found that salivary microbiomes with larger proportions of *Prevotella* and *Veillonella* were associated with periodontitis, whereas larger proportions of *Neisseria*, *Haemophilus*, and *Porphyromonas* were associated with periodontal health in the Japanese population, but was not the case in the Korean population^31,32^. To validate the reliability of the T2DM group’s data, high quality sequencing data from the study by Sabharwal et al. was used as a validation set^22^. The performance was relatively lowered probably due to the influence of geographical and racial factors, however, we thought that the accuracy was acceptable. Thus our current study may not be applicable in non-Chinese population and further multicenter clinical study is needed to verify the prediction accuracy of the established random forest models.

To conclude, an increase in saliva microbial diversity was observed in periodontitis patients with or without companion T2DM. Active glycemic control could lead to a change in oral microbial communities resembling the healthy population, which is beneficial to improve the localized conditions of DAP patients. The saliva microbiota might effectively predict the risk of developing T2DM in periodontitis patients.

## Methods

### Study design

A total of 133 participants were recruited from the Stomatologic Hospital & College of Anhui Medical University (Anhui, China). The inclusion criteria included: (1) patients diagnosed with periodontitis (P group); type-2 diabetes mellitus (T2DM group); periodontitis in combination with T2DM (DAP group); patients in the DAP group treated with metformin (Met group); healthy subjects without periodontitis, T2DM, and other systemic diseases during health-checkups within 1 year of this study (Health group); (2) patients aged from 18 to 80 years old upon enrollment; and (3) patients who provided signed informed consent. The exclusion criteria included: (1) patients treated with antibiotics within 3 months or those that received periodontal treatment within 6 months; (2) pregnant or lactating women; (3) current smokers; (4) patients with other systemic diseases (e.g., cardiovascular, pulmonary, liver and/or cerebral diseases). The study was approved by the Ethics Committee of the Anhui Medical University for Nationalities (NO.20180163) and conducted in accordance with the Declaration of Helsinki. This trial has been registered in the Chinese Clinical Trial Registry (Registration number: ChiCTR1800015652).

A diagnosis of periodontitis was made according to the criteria of the 1999 International Classification for periodontal diseases^33^. In brief, periodontitis patients had a PD of ≥ 5 mm and a CAL of ≥ 3 mm. T2DM was determined by a fasting blood sugar of > 7.0 mmol/L and an HbA1c concentration of > 7%, as stated by the American Diabetes Association in 2018^34^. Patients were classified into the DAP group when both periodontitis and T2DM was present. DAP patients who received metformin for at least 6 months and achieved glycemic control (HbA1c < 6.5%, without any companion diseases), were classified into the Met group.

### Clinical assessment and sample collection

All the participants were referred to a specialized dental clinic for periodontal assessment. The BMI, age, and gender were recorded. A full-mouth periodontal examination was conducted by an experienced dentist. Periodontal disease was assessed by determining the PD, CAL, BI, GI, and PLI, which were measured at six sites (i.e., mesiobuccal, buccal, distobuccal, distolingual, lingual, and mesiolingual) according to the Community Periodontal Index^35^. The determinations were performed by a UNC-15 probe and rounded off to the nearest millimeter.

Saliva samples were collected from all the participants before clinical evaluation. All subjects rinsed with tap water (10 mL) for 30 s and expectorated before saliva collection. Unstimulated whole saliva was collected as described by Navazesh^36^. Subjects were asked to avoid oral hygiene measures (i.e., flossing, brushing, and mouth rinsing), eating, drinking, or gum chewing 1 h before saliva collection. Subjects expectorated at least 2 mL of unstimulated whole saliva into sterile tubes. Saliva samples were frozen at − 80 °C until analysis.

### DNA extraction, library construction, and sequencing

DNA extraction and purification were performed by the QIAamp DNA Stool Kit (Qiagen, Valencia, CA, USA) according to the manufacturer’s instructions. The V3-V4 region of the 16S rRNA was amplified with the polymerase chain reaction (PCR). In brief, PCR was performed with the diluted genomic DNA as the template using specific primers with barcodes, Phusion High-Fidelity PCR Master Mix with GC Buffer (New England Biolabs, UK), and high-efficiency high-fidelity polymerase. The forward primer sequence was 3′-CCTAYGGGRBGCASCAG-5′ (341F), and the reverse primer sequence was 3′-GGACTACNNGGGTATCTAAT-5′ (806R). The TruSeq DNA PCR-Free Sample Preparation Kit (Illumina, San Diego, CA, USA) was used for library preparation. The constructed library was quantified by Qubit and qPCR for quality control and sequenced with the HiSeq2500 PE250 platform (Illumina). The PCR products were also sequenced with the HiSeq 2,500 PE250 platform (Illumina).

### Sequencing data analysis

Data analysis was performed by automated streamlined software (Torrent Suite software version 5.0). All sequence data were clustered into OTUs at a 97% similarity threshold by vearch v2.15.10_linux_x86_64 and usearch10.0.240_i86linux32^37^. Alpha-diversity indices were estimated based on observed-OTUs, Chao1, Shannon, and Simpson values^38,39^. The total number of OTUs in each group was displayed by Venn diagrams (created using online tools available at https://jvenn.toulouse.inra.fr/app/example.html). Alpha-diversity indices, including Chao1, Simpson, Shannon, observed-OTUs, and Faith’s Phylogenetic Diversity (Faith-PD), were calculated using QIIME2 (version 2020.2)^40^. The top 30 core microbiomes at OTU, genus, and species levels and the top ten core microbiomes at the phylum level with the highest relative abundance in each group were displayed in bar charts (R package, ggplot2, 3.3.1). Principal component analysis (PCA) and Principal coordinate analysis (PCoA) were performed using R version 4.0.0. PCoA was used to calculate the Bray–Curtis, Jaccard, unweighted UniFrac, and weighted UniFrac distances. The common microbiomes at OTU, phylum, genus, and species levels between groups were illustrated by a heatmap (R package, pheatmap, version 1.0.12).

Relative abundance analysis of OTUs was performed using Deducer (R package, version 0.7–9). Permutation testing was performed with a replacement number of 10,000 times. The identified microbiota with a relative abundance difference more than three times between groups and an abundance greater than 10^–4^ within the group was expressed in bar charts.

Correlation testing between the clinical index and salivary microbiome was performed using Spearman correlation analysis. Microbiomes with a relative abundance of < 0.02% were excluded from the analysis. A relationship graph was generated with Cytoscape software (version 3.6.1) to illustrate the microbiome with correlation values of ≥ 0.4.Random forest analysis (R package, random Forest, version 4.6–14) was used to establish classification models for the diagnosis of periodontitis and T2DM. The predictive performance of the classification models was analyzed by receiver operating characteristic curves (R package, pROC, version 1.16.2).

The clinical parameters were analyzed by SPSS 23 software (IBM Corp., Armonk, NY, USA). PERMANOVA with 10,000 permutations was performed on the unweighted UniFrac distances using the adonis function in the vegan package (version 2.5–6) in R^41^. The taxonomic abundance in the cross-sectional analysis was determined using the Wilcoxon rank-sum test. An independent t-test was performed to compare the difference in clinical factors between disease groups. For factors that satisfied the conditions of normality and homogeneity of variance, PERMANOVA analysis was used to compare the difference between groups.

## Data availability

Sequencing data can be found at the submission portal of NCBI (https://www.ncbi.nlm.nih.gov/bioproject/PRJNA601054).

## Supplementary information

Supplementary figures.
