# Supplementary material for: Alteration of salivary microbiome in periodontitis with or without type-2 diabetes mellitus and metformin treatment
Source: Sci Rep. 2020 Sep 21;10:15363. doi: 10.1038/s41598-020-72035-1 (PMC7506544; doi:10.1038/s41598-020-72035-1)
Supplement: Supplementary file 1 — Supplementary figures. [file 41598_2020_72035_MOESM1_ESM.pdf]

Title: Alteration of salivary microbiome in periodontitis with or without type-2 diabetes mellitus and metformin treatment

Author list: Xiaoyu Sun, Meihui Li, Li Xia, Zhaohui Fang, Shenjun Yu, Jike Gao, Qiang Feng*, Pishan Yang*

Figure S1. Phylogenic diversity of the oral microbiota in different groups

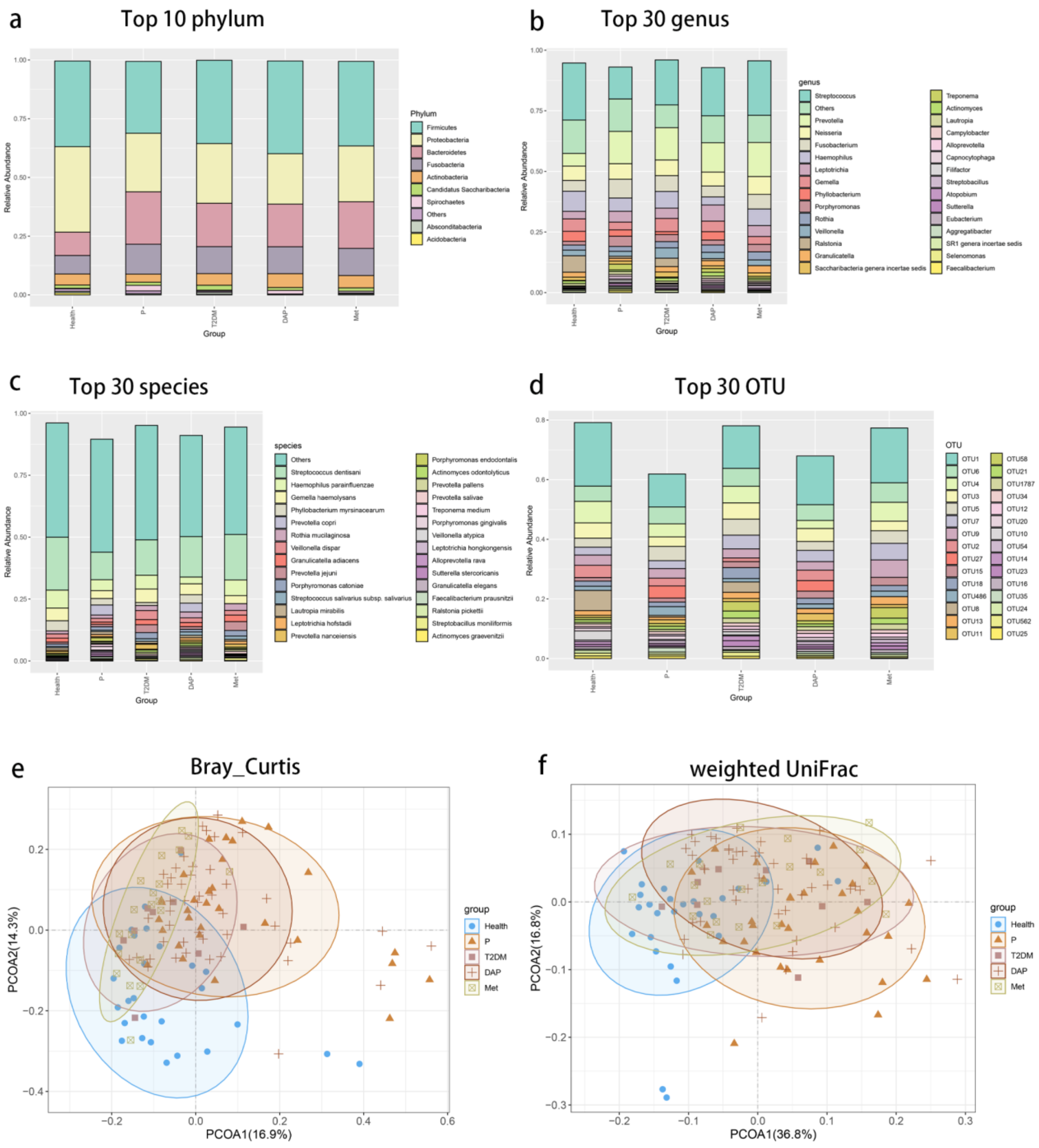

(a) The top 10 phyla, (b) top 30 genus, (c) top 30 species, and (d) top 30 OTUs in each group in terms of relative abundance. The beta-diversity of each group as shown by PCoA analysis according to (e) Bray-Curtis index and (f) weighted-UniFrac index.

Figure S2. The “core microbiome” of all groups.

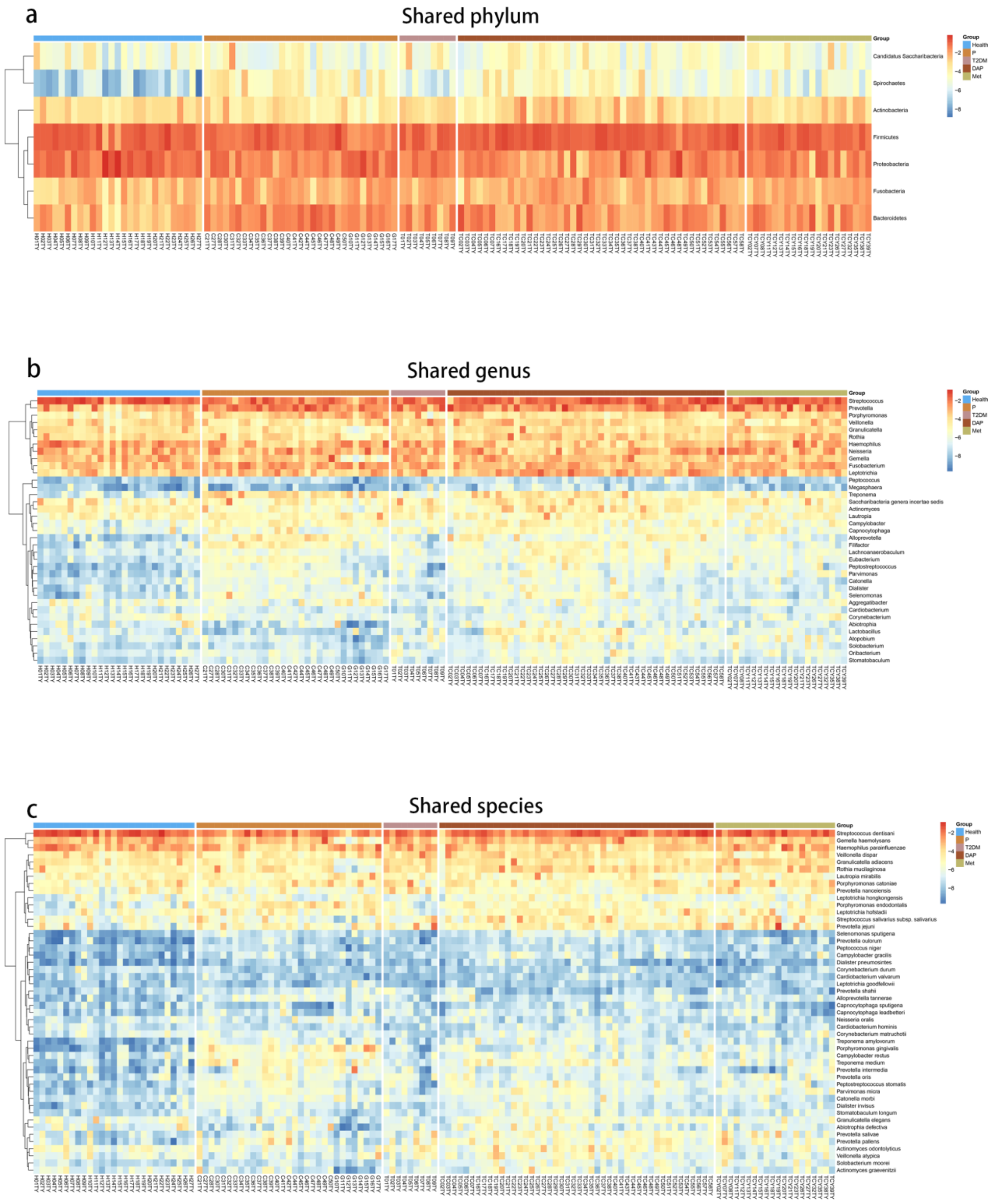

(a) All shared phylum between groups. (b) All shared genus between groups. (c) All shared species between groups.

Figure S3. The relative abundance of oral microbiota between groups

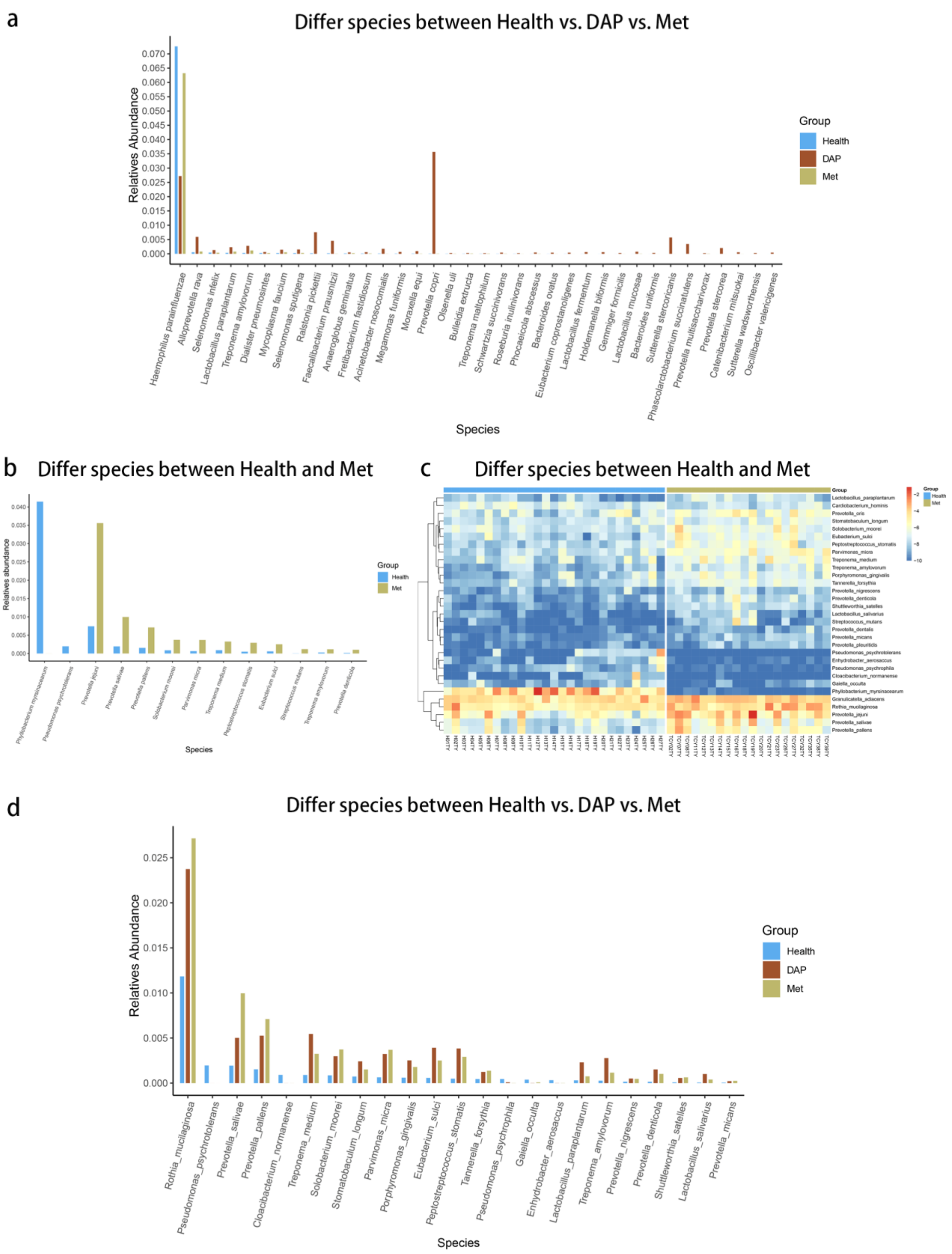

The difference in relative abundance of oral microbiota between the (a) Health versus DAP and DAP versus Met and (d) Health versus DAP and Health versus Met were listed in a bar chart. (b) Bar chart showing the relative abundances of oral microbiota between the Met and Health groups; (c) Heatmap showing the difference in relative abundance of oral microbiota betweenthe Met and Health groups.

Figure S4. Association between microbial communities and clinical parameters

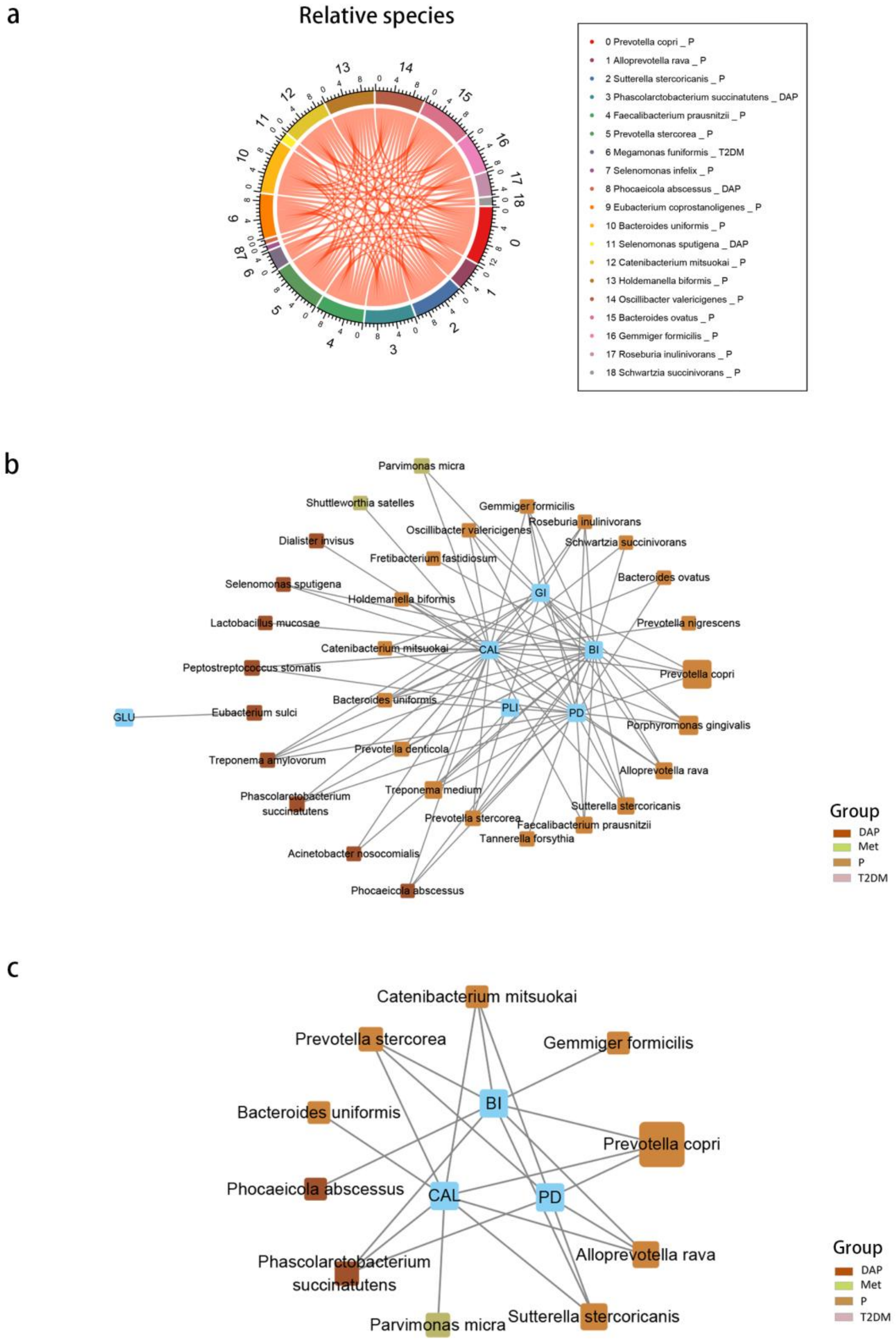

(a) All the correlation between microbial communities at species level (cor≥0.8). The association between microbial communities and clinical parameters with (b) correlation value≥0.5 and (c) correlation value≥0.6.

Figure S5. Selected core saliva microbiota for establishing the classification models

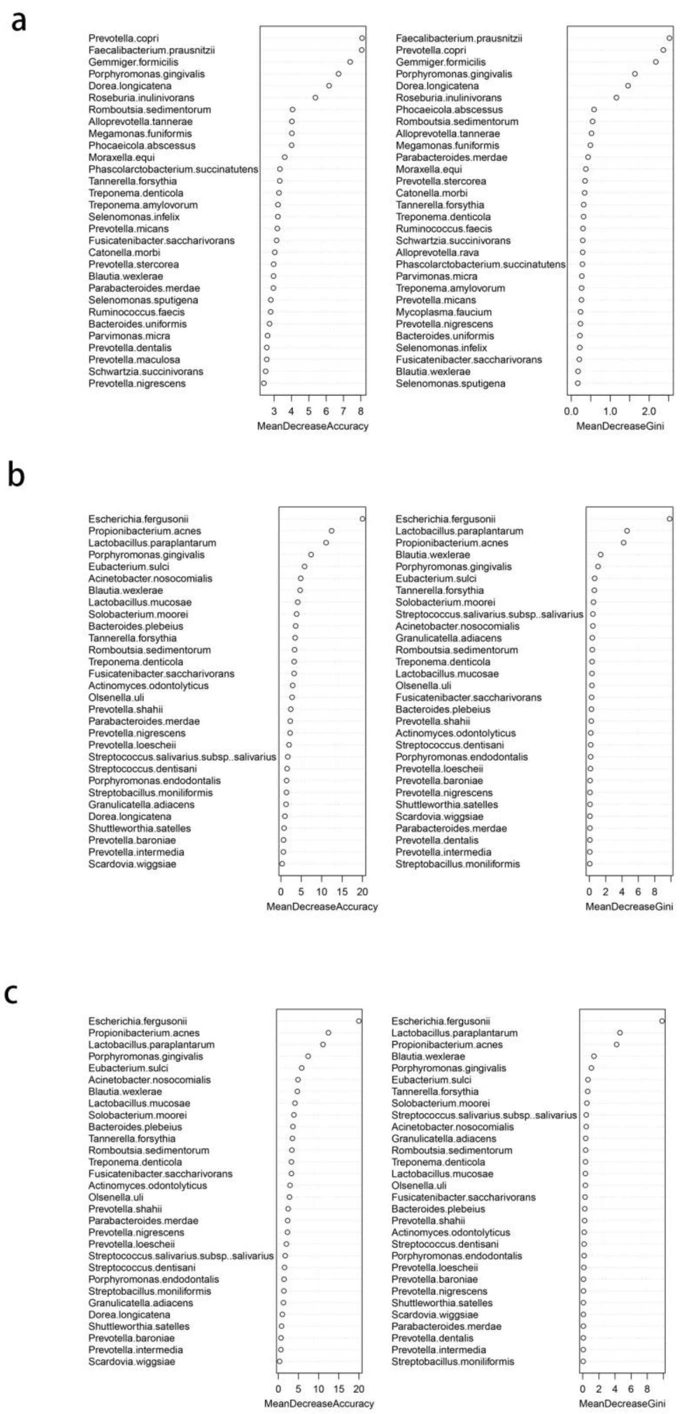

Dot plots showing the selected core saliva microbiota for establishing the (a) P versus Health, (b) P versus DAP, and (c) T2DM versus DAP classification models sorted according to the importance.

Figure S6. Prediction performance of the classification model in the validation set.

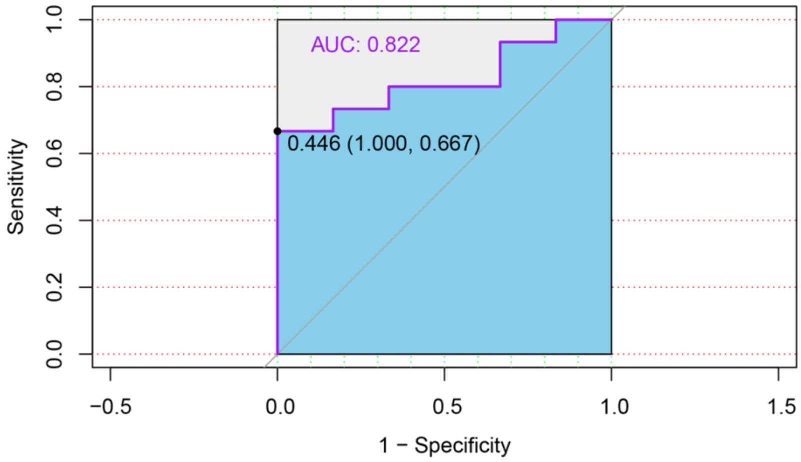

ROC curve of the selected core microbiota in differentiating between the T2DM group and the DAP group.
